# Potential Benefits of Adding Alendronate, Celecoxib, Itraconazole, Ramelteon, and Simvastatin to Endometrial Cancer Treatment: The EC5 Regimen

**DOI:** 10.3390/cimb47030153

**Published:** 2025-02-26

**Authors:** Richard E. Kast

**Affiliations:** IIAIGC Study Center, Burlington, VT 05408, USA; richarderickast@gmail.com

**Keywords:** celecoxib, endometrial cancer, itraconazole, ramelteon, repurposing

## Abstract

Metastatic endometrial cancer continues to be a common cause of death as of 2024, even after maximal use of all currently available standard treatments. To address this problem of metastatic cancer generally in 2025, the drug repurposing movement within oncology identifies medicines in common general medical use that have clinical or preclinical experimental data indicating that they interfere with or inhibit a specific growth driving element identified in a given cancer. The drug repurposing movement within oncology also uses data from large scale in vitro screens of thousands of drugs, looking for simple empirical growth inhibition in a given cancer type. This paper outlines the data showing that five drugs from general medical practice meet these evidence criteria for inhibition of endometrial cancer growth, the EC5 regimen. The EC5 regimen uses the osteoporosis treatment drug, alendronate; the analgesic drug, celecoxib; the antifungal drug, itraconazole; the sleep aid, ramelteon; and the cholesterol lowering drug, simvastatin. Side effects seen with these drugs are usually minimal and easily tolerated by patients.

## 1. Introduction

The following regimen, EC5, has been designed as an adjunct to current treatments for metastatic endometrial cancer (EC). EC5 uses five drugs from general medical practice, not traditional oncology drugs, to augment current traditional cytotoxic or kinase inhibitor drugs for treating EC. EC5 uses the osteoporosis treatment drug, alendronate; the analgesic drug, celecoxib; the antifungal drug, itraconazole; the insomnia drug, ramelteon; and the cholesterol lowering drug, simvastatin. Table 1 lists the EC5 drugs with their general medical use and proposed use in EC. Table 2 lists their target doses and common side effects. How each of these five drugs intersect with and inhibit the cancer growth mechanisms of EC is detailed in the respective drug’s section below.

Repurposing non-oncology drugs for adjunctive cancer treatment is an area of rapid expansion as oncology recognizes their utility [1,2,3,4,5,6]. Cancers, generally, and EC, specifically, use normal mammalian physiology systems to grow and metastasize. They pathologically enlist fundamentally normal physiological homeostatic mechanisms. The mutated, cancer-specific DNA abnormalities drive malignant growth, but the mutated systems must use ordinary, ubiquitous growth systems to create the malformed, dyscontrolled cancer cells. General medical practice has evolved several hundred safe and effective generic drugs to treat a wide variety of diseases where these normal physiological systems cause disease. We can use these non-oncology drugs to defeat or inhibit the body’s normal physiology systems that become co-opted by the malignant cells.

As of 2024, EC incidence and mortality have been increasing [7]. Rising incidence of obesity and prolonged unopposed estrogen exposure are suspected as contributory to that increase [7]. ECs are biologically, histologically, clinically, and mutationally diverse. EC commonly metastasizes to local lymph nodes, lungs, and local peritoneum [8]. Type 2 diabetes, obesity, hypertension and arteriosclerosis are risk factors for EC.

Several classification systems co-exist as of 2024. Prior classification divided EC into Type I, which are commonly associated with unopposed estrogen stimulation with low-grade cells, have a favorable prognosis, and are endometrioid adenocarcinomas. Type II are usually not estrogen driven, are endometrioid, serous clear cell or undifferentiated, and have an unfavorable prognosis. EC is further divided by stage, Stage 1 describing tumors with minimal myometrial invasion; Stage 2, tumors extending into the cervix; Stage 3, tumors which have regional spread beyond the uterus; Stage 4, invasion of bladder, bowel, or tumors with distant metastases. As invasion of the myometrium becomes progressively deeper, lymph node metastases become more likely [7,8,9,10,11,12].

Much of the preclinical data on EC do not specify or characterize the EC subtype studied. Therefore, most of the data on the EC5 drugs’ inhibition of EC growth are subtype agnostic.

The 2020 WHO classification divides EC into endometrioid carcinoma, serous carcinoma, clear cell carcinoma, carcinosarcoma, and undifferentiated/dedifferentiated carcinoma. Several rare subtypes cannot fit into these categories [7,8,9,10]. EC has a relatively low (~70%) concordance rate among pathologists when assessing H&E-based histotype and grade of EC.

## 2. The EC5 Drugs

Candidate drugs were chosen based on two criteria—strength of preclinical evidence for EC growth inhibition and low side effect burden imposed by a candidate drug as established by its use in day-to-day general medicine use.

### 2.1. Alendronate

Alendronate belongs to the bisphosphonate class of drugs used to treat Paget’s disease of bone, myeloma, bone metastases, and treat or prevent osteoporosis [13,14,15,16]. Alendronate is highly bone avid, inhibits farnesyl pyrophosphate synthase, inhibits expression of several matrix metalloproteinases (MMP), and has constructive effects on lymphocytes. To what degree these disparate effects are related to or secondary to a unitary effect remains unknown.

Alendronate is a member of the nitrogen-containing bisphosphonate group (the others are risedronate, ibandronate, and zoledronic acid). Alendronate inhibits osteoclast activity secondary to its inhibition of farnesyl pyrophosphate synthase [17,18,19].

Alendronate collects at the margins of an osteolytic malignant cell metastasis [20,21].

Several examples of other pharmaceutical nitrogen containing bisphosphonates that inhibit farnesyl pyrophosphate synthase will reduce stem cell functions in models of: glioblastoma [22], pancreatic ductal cancer [23], ovarian epithelial cancer [24], normal marrow CD133+ stem cells [25], colon adenocarcinoma [26], acute myeloid leukemia [27,28], and others.

Atypical femoral fracture and osteonecrosis of the jaw are risks that come with alendronate. This risk rises with high cumulative doses and after years of use. Of the bisphosphonates, alendronate has the least anti-bone resorption activity but does reduce fracture incidence in those with, or at risk of developing, osteoporosis. Bone resorption inhibition potencies are alendronate < ibandronate < risedronate < zoledronic acid [29]. Estimates vary, but about 1 in 4000 long term alendronate users will experience osteonecrosis of the jaw [30].

#### 2.1.1. Alendronate, Empirical Data

Multiple independent studies examining different populations have found a consistent association between lower risk of EC in those taking bisphosphonates, and this risk reduction was not small: 25% or more in those with multi-year bisphosphonate use [31,32,33,34,35,36]. In a study of 29,000 women, the incidence rate for endometrial cancer among those on the nitrogen-containing bisphosphonates like alendronate was 8.7 per 10,000 person-years versus 17.7 per 10,000 person-years among never-exposed women [34]. Similar risk reduction was seen in epidemiological study of some (but not all) other common cancers [36,37,38]. In vitro and preclinical studies of alendronate have shown a cancer-specific cytotoxicity [39,40].

A series of papers showed growth inhibition specifically by alendronate across many of the common cancers. Table 3 lists a few representative papers from that research database.

#### 2.1.2. Alendronate, MMP

Alendronate inhibited MMP-3, -12, -13, and -20, as well as MMP-1, -2, -8, and -9 in vitro at therapeutically attainable concentrations that are non-cytotoxic to nontransformed cells [46,52,53,54,55]. Of the two dozen MMPs, MMP-2, and MMP-9 are the most prominent in EC myometrial invasion [56,57,58,59,60]. MMP-2 and MMP-9 have several roles in furthering malignant growth across the common cancers [61,62].

#### 2.1.3. Alendronate, Rho

Alendronate inhibits lysophosphatidic acid-induced migration of human ovarian cancer cells by attenuating the activation of the small GTPase Rho [63,64,65]. Alendronate inhibits Rho in several different contexts and in different cell systems. Rho inhibition by alendronate interferes with vessel endothelium function, limiting tumor angiogenesis [63,64,65,66,67,68].

A significant proportion of cases of EC have a mutation-driven overactivation of Rho as part of their suite of pathophysiologic disruptions [69,70,71,72,73,74].

#### 2.1.4. The Problem of Alendronate, Gamma–Delta T Cells

The dataset on gamma–delta T cells in cancer treatment is problematic. Indeed, the idea of using alendronate as an adjunct in treating any cancer is problematic on several grounds, as discussed throughout this paper.

We have acquired good evidence that higher numbers of gamma–delta T cells benefit patients, and we have acquired good evidence that higher numbers of gamma–delta T cells do not benefit patients. There is clearly something important missing in our understanding of gamma–delta T cells in cancer.

Gamma–delta T cells (gamma–delta T lymphocytes) are a subset of T lymphocytes which constitute 1–10% of circulating T cells in humans [75,76,77,78]. In EC, those with elevated gamma–delta T cells levels experienced longer survival [79]. This association of higher gamma–delta T cells infiltrating human tumors was also found in studies of breast cancer [80], colon adenocarcinoma [81], melanomas [82], head and neck squamous cell carcinoma [83], and hepatocellular carcinoma [84].

However, several dozen early phase clinical cancer trials directed at increasing gamma–delta T cell numbers or increasing their activity have failed to show benefits as of the end of 2024 [85,86,87,88,89]. The point of failure, origin of discrepancy between epidemiological and histological data, and failure to benefit clinically in these studies remains unknown.

Gamma–delta T cells express T cell receptors gamma (γ) and delta (δ) chains. gamma–delta T cells have antigen recognition sites, secrete several cytokines, and express granzymes and perforin for target cell lysis that does not depend on target cell MHC expression [76,77,85,88,90]. Evidence links gamma–delta T cells with a specific role in destroying malignant cells [89,91,92]. Uniquely, unlike other lymphocytes, gamma–delta T cells do not require antigen presenting cells and do not read HLA before target antigen bearing cell killing [77,93].

**Figure 1:**
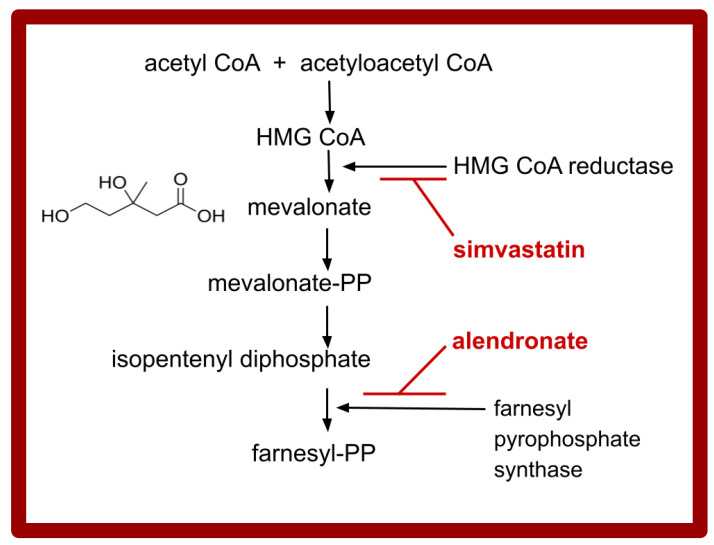
Many intermediate steps have been omitted from this schematic overview of the locus of action for simvastatin and for alendronate. Farnesyl diphosphate synthase is synonymous with farnesyl pyrophosphate synthase. PP, pyrophosphate; figure adapted from refs. [17,18,19].

Gamma–delta T cells directly recognize and kill transformed cells independently of HLA-antigen presentation. Gamma–delta T cell receptors sense intracellular phosphorylated metabolites, which accumulate in cancer cells by inherent malignant cell dysregulation, hypermetabolism, or as a consequence of mevalonate inhibition. See the simplified schematic Figure 1, of the mevalonate pathway and locus of alendronate action. Gamma–delta T cells target cell killing is performed by the release of granzyme and perforin, or by the production of IFNγ or TNFα to amplify the immune response [87,88,94].

Alendronate and the other pharmaceutical nitrogen containing bisphosphonates activate gamma–delta T cells to acquire effector functions [77,95,96,97,98]. However, in pharmacodynamic analyses across a variety of cancers, though patients showed significant in vivo activation (increased interferon-γ production) and expansion of gamma–delta T cells by zoledronic acid plus IL-2, no clinical benefit was noted [95].

Ex vivo expansion in gamma–delta T cells from multiple myeloma patients by exposure to IL-2 and zoledronic acid, when reinfused back into donors, resulted in stably increased effector memory gamma–delta T cells in all patients [97]. Patient survival was not reported. Children with advanced neuroblastoma who had exhausted all other treatment options had half the number of peripheral gamma–delta T cells compared to normal age matched controls [98]. Their gamma–delta T cells increased three- to ten-fold after treatment with zoledronic acid and IL-2. Their NK cells also increased. There was no clear clinical benefit. In a ten-patient study in advanced widely metastatic breast cancer, four out of four patients that had increased gamma–delta T cells after zoledronic acid plus IL-2 survived 12 months or longer, while six out of ten who showed no gamma–delta increase died before 12 months [99].

However, in postmenopausal women with osteoporosis receiving zoledronic acid treatment, after one year circulating numbers of gamma–delta T cells decreased, while NK cells increased [100].

#### 2.1.5. Simvastatin and Alendronate

Statins comprise a group of drugs that are used to treat hypercholesterolemia by inhibiting 3-Hydroxy-3-methylglutaryl (HMG)-CoA reductase. A series of studies have documented the additive effects of blocking the mevalonate pathway at two points, as depicted in Figure 1. The common statin drug used to treat hypercholesterolemia, simvastatin, has an interesting and important database on its augmentation of alendronate effects, in settings of both bone maintenance and in the anticancer role.

Of the statins approved for use in humans to lower cholesterol, the lipophilic statins (atorvastatin, fluvastatin, lovastatin, pitavastatin, simvastatin) increased threefold the antibody titre after immunization in a murine model, compared to the hydrophilic statins (rosuvastatin and pravastatin) [17]. This increase in antibody titre by alendronate and simvastatin has not been repeated or investigated further.

Many preclinical studies have shown a specifically alendronate-driven increase in gamma–delta T cells [101,102,103]. Alendronate induced activation and expansion of normal human gamma–delta T cells, triggering their differentiation to an effector/cytotoxic phenotype [103,104]. Multiple other studies have shown expansion in gamma–delta T cells by other bisphosphonates (like zoledronic acid) [105,106,107,108,109]. This dataset altogether implies bisphosphonates’ provocation of an increase in gamma–delta T cells as a drug class effect.

Either alendronate or simvastatin slightly enhanced bone area within an experimentally created femur defect in mice. The combination of both resulted in a greater bone area than either one individually [110]. These findings were replicated by others [111]. The same combination, alendronate and simvastatin, increased bone mass in ovariectomized rats [112]—more than either alone, but less than additive. Both alendronate and simvastatin individually increased bone mass and bone density in ovariectomized rats [113]. The combination was not tested in this work. Elderly patients with endometrial cancer taking a statin did not experience longer survival compared to those not on a statin [114]. Intra-alveolar simvastatin post-tooth extraction was effective and safe for preserving alveolar bone, with varied carriers and no significant adverse effects [115].

Combining other bisphosphonates with other statins have produced similar effects in various settings greater than either did individually [116,117,118,119,120].

A meta-analysis of 14 studies encompassing 1456 people with Type 2 diabetes and osteoporosis concluded that alendronate combined with atorvastatin is more effective than alendronate alone [121].

A sequential blockade of the mevalonate pathway by alendronate and simvastatin was synergistic in inhibiting some prostate cancer cell lines but not others [122]. Mixed micelles of D-α tocopherol polyethylene glycol 1000 succinate and Poloxamer-407 containing alendronate plus simvastatin exhibited profound growth arrest in the ng/mL range to a variety of cancer cell lines in vitro [123].

A study of alendronate and simvastatin was synergistic in reducing bone loss caused by dexamethasone in rats [124]. In rheumatoid arthritis treated with prednisone, less bone loss occurred in those taking a bisphosphonate and a statin compared to those taking only a bisphosphonate [125].

Inhibiting the mevalonate pathway enhances antigen-specific anti-tumor immunity, inducing both Th1 and cytolytic T cell responses [120].

Only minor increases in circulating gamma–delta T cells were observed after in vitro pamidronate or zoledronic acid in a group of advanced cancer patients [126]. Adding oral ibandronate to standard treatment had no statistically significant effect on overall survival in breast cancer [127]. A slight trend toward a longer survival was only seen in patients <40 and >60 years old, and did not reach statistical significance.

### 2.2. Celecoxib

Celecoxib is a pharmaceutical analgesic drug that has two direct prominent physiological attributes: (i) it inhibits COX-2 and (ii) it inhibits several carbonic anhydrases (CA). Potentially useful consequences for EC treatment follow from these two attributes.

In addition to widespread use as a simple, low-risk, and non-opiate analgesic, celecoxib has developed a large research database on cancer growth inhibition [128,129,130,131,132,133,134]. Hundreds of published research papers have demonstrated increased COX-2 expression in malignant tissue.

#### 2.2.1. Celecoxib, COX-2

Higher COX-2 overexpression is significantly associated with poorer prognosis in EC compared to EC cases with lesser COX-2 overexpression [135,136,137,138]. Others have found that, though COX-2 is overexpressed in EC tissue, the degree of overexpression was not associated with survival duration [139]. Immunohistochemical COX-2 staining increases with increasing EC stage [140]. As we see with several other markers, COX-2 immunohistochemical staining becomes heavier as ECs progressively invade the myometrium [141]. Paclitaxel resistant EC cells express greater COX-2 amounts than the paclitaxel sensitive EC cells do [142].

It is also important to note that COX-2 expression and aromatase expression are correlated in EC [143]. This would suggest that celecoxib lowers IL-6 in EC by interrupting this feedback cycle.

The malignant cells of EC can induce the local non-transformed endometrial cells to synthesize increased COX-2 [144].

#### 2.2.2. Celecoxib, CA-II, CA-IX, CA-XII

Celecoxib inhibits CA isoforms II, IV, IX, and XII [131,145,146,147]. CA-IX and XII are transmembrane; CA-II is soluble.

As an example of celecoxib’s CA inhibition profile compared to the standard CA inhibitor acetazolamide, Sethi et al. [147] found

- acetazolamide   IC50 at CA-II = 12, CA-IX = 25, CA-XII = 6
- celecoxib            IC50 at CA-II = 21, CA-IX = 16, CA-XII = 18
- dorzolamide    IC50 at CA-II = 9, CA-IX = 52, CA-XII = 4

**Figure 2:**
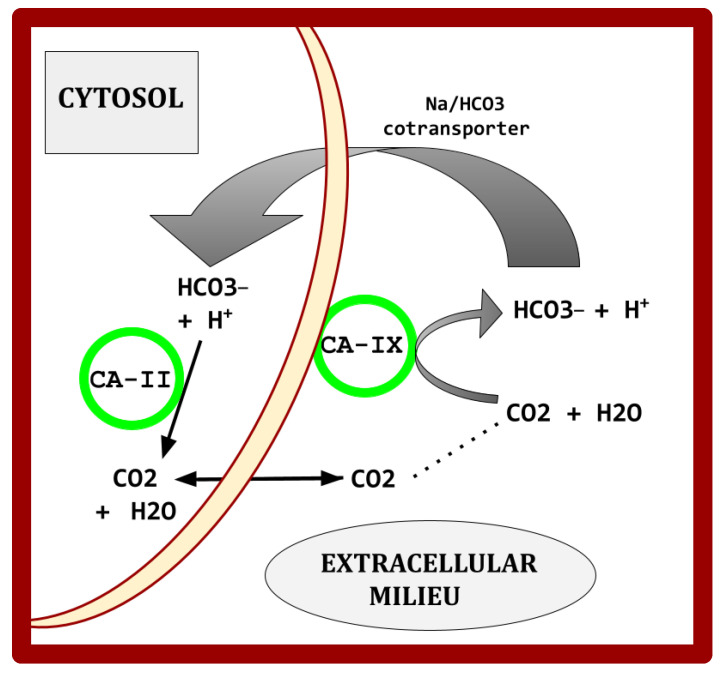
Simplified basic diagram on how two carbonic anhydrases, CA-II and CA-IX, aid in milieu acidification and in maintenance of cytosolic high pH in cancer. CA-II is soluble, found in the cytosol. CA-IX and CA-XII are external and membrane bound.

Elevated CA isoforms are characteristic of EC tissues [148]. Overexpressed in EC, CA-II and -IX regulate the pH of the EC tumor microenvironment, as schematically illustrated in Figure 2, preventing the lowering of intracellular pH that would otherwise occur in EC [149,150]. As in Figure 2, cytosol CA-II functions in partnership with membrane-bound CA-IX.

Elevated CA isoforms -IX and -XII are core features also found across the common cancers [151,152,153,154,155,156,157,158]. Figure 2 shows the basic mechanism by which CAs participate in creating the characteristic acidic peritumoral milieu and maintenance of intracellular alkalinization in malignancy.

As examples, CA-IX and/or CA-XII are elevated in, worsen survival, and contribute to malignancy grade in many cancers: acute myelogenous leukemia [159,160], bladder urothelial cancer [161,162,163,164,165,166], breast [167], esophageal [168,169], gastric [169,170], glioblastoma [171], hepatocellular [172], Hodgkin’s lymphoma [173], laryngeal [174], nasopharyngeal [175,176], non-small cell lung [177], oral squamous cell [178], osteosarcoma [179], pancreatic ductal [180], endometrium [148], and thyroid [181].

This dataset leads to the conclusion that CA-IX and/or CA-XII tends to be one of the many links in mediating or facilitating malignant cell behaviors across many of the common cancers. This finding is not universal, however.

Celecoxib, in adequate doses, may hobble EC growth by limiting EC’s ability to cope with its increased metabolism-related acid production. An indication that this is occurring is the findings of glioblastoma, where resistance to temozolomide is overcome by CA-XII inhibition [182,183].

#### 2.2.3. Celecoxib, IL-6

Of the many cytokines elevated in and facilitating EC, IL-6 plays an important role [184,185,186]. This section presents data indicating that lowering IL-6 improves EC prognosis and that celecoxib mediates this lowering.

Clinical use of celecoxib reduced the elevated circulating IL-6 found in patients with ankylosing spondylitis [187], pancreatitis [188], major depression [189], heavy tobacco smoking [190], frailty of age [191], knee osteoarthritis [192], and inflammatory arthritis [193].

IL-6 is the quintessential pleiotropic cytokine. Its signaling is ubiquitous and central to mammalian physiology. But, what it does cannot be simply stated. IL-6 can be pro- or anti- inflammation, depending on circumstances [194,195].

**Figure 3:**
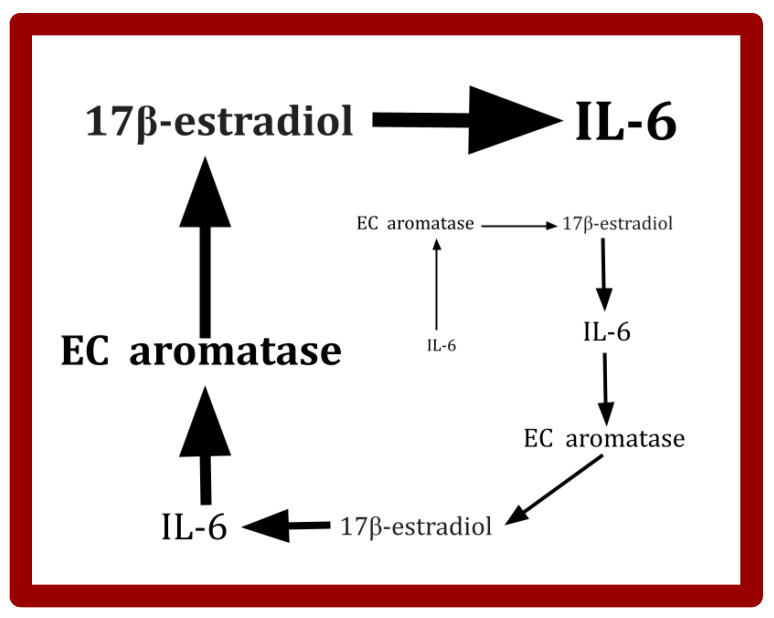
A simplified schematic of an amplifying IL-6 feedback loop active in EC where IL-6 induces an increase in EC cell aromatase that leads to increased IL-6, and so on [196,197,201]. Note also that COX-2 expression and aromatase expression are correlated in EC [143]. This would suggest that celecoxib lowers IL-6 in EC by interrupting this feedback cycle. Increasing font size represents an increasing amount. Several intermediate steps or links in this feedback loop are omitted from the schematic. Nonmalignant EC stromal cells (fibroblasts and non-transformed myometrial cells) participate in this loop [202,203]. Likely on theoretical grounds are the existence of counterbalancing counterregulatory forces that would dampen this amplifying cycle, preventing runaway amplification.

Excess IL-6 is one of the drivers of EC invasion and migration [194,195,196,197,198]. An amplifying feedback loop between IL-6 and aromatase exists, as schematically diagrammed in Figure 3 [199,200]. Omitted from Figure 3 are several intermediate links in this amplification chain. Also omitted are counterregulatory inhibiting elements that will stabilize IL-6 at some point.

Epidemiological studies show that women with higher circulating IL-6 are at a statistically higher risk of developing EC [201,204,205]. EC cases with greater mRNA for IL-6 experience shorter mean survival compared to those with low IL-6 mRNA [206]. Serum IL-6 increases as EC progressively invades the myometrium [185,207]. Particularly high levels of IL-6 are seen in serous EC with papillary histology. These are aggressive and chemotherapy-resistant tumors [208].

The EC cell subpopulation that exhibits stem cell characteristics of high ALDH expression, high clonogenicity, and low cell numbers needed to transplant tumors, have increased IL-6 receptor proteins compared to EC cells without these stem attributes [209].

Non-transformed, otherwise normal fibroblasts resident within an EC secrete higher levels of MCP-1, IL-6, IL-8, and RANTES than normal uterine fibroblasts do [210,211]. The trigger or signaling system driving these otherwise normal fibroblasts to secrete abnormal amounts of these cytokines is unknown, but presumably the EC malignant cells perform the recruiting to serve their growth needs.

We know that intratumoral non-transformed fibroblasts support and promote the growth of the transformed malignant cells of cancers generally [202,203,212,213]. Fibroblasts fulfill many crucial physiological functions crucial for proper homeostatic functioning of all body organs: scaffolding, providing trophic growth signals, recruiting bone marrow cells for angiogenesis, shaping or inhibiting lymphocyte centered immune response, and others. In EC specifically, though enlisted to promote cancer growth, EC resident fibroblasts are essentially normal fibroblasts responding normally to their normal directing signaling systems, even though the net effect of which promotes EC growth [214,215,216,217]. In vitro work has shown that celecoxib can diminish cancer tissue resident non-transformed fibroblasts’ trophic function to the malignant cell population [218,219,220].

### 2.3. Itraconazole

Itraconazole is commonly used in general medical practice to treat superficial, as well as serious, systemic fungal infections [221,222,223]. Ergosterol is the predominant sterol in fungal outer membranes [224]. Itraconazole inhibits ergosterol synthesis, thereby interfering with fungal cell wall synthesis. It is also a strong inhibitor of mammalian Hedgehog [Hh] signaling [225,226,227].

Itraconazole has a volume of distribution of 11 L/kg, indicating higher tissue concentration compared to circulating levels.

Itraconazole is currently undergoing a renaissance as a repurposed drug treatment adjunct across a variety of common cancers [228,229,230,231,232].

There are only two studies testing itraconazole for activity specifically in inhibiting EC. Itraconazole inhibited EC cell survival and migration in vitro by reducing Hh signaling [233,234]. An antifungal drug closely related to itraconazole and ketoconazole, was shown this year to inhibit EC’s angiogenesis [235].

Counterbalancing this paucity of direct evidence for itraconazole’s specific cytotoxicity or ability to inhibit growth in EC are dozens of studies attesting to intraconazole’s robust inhibition of growth across a wide variety of other common cancers. Some representative examples of this are itraconazole’s growth inhibition of acute lymphocytic leukemia [234], acute myelogenous leukemia [236,237], basal cell carcinoma [238,239], glioblastoma [240,241,242], melanoma [243], mesothelioma [244], mycosis fungoides [245], osteosarcoma [246], rhabdomyosarcoma [247], and cancers of the breast [248,249], colon [250], endometrium [233,234], esophagus [251,252], head and neck [253], liver [254], lung [255,256,257,258], ovary [259,260], pancreas duct [261,262,263,264], prostate [265,266,267], and stomach [268,269].

### 2.4. Ramelteon

Ramelteon is an older generic melatonergic agonist marketed to improve sleep [270,271]. It is a full agonist at melatonin’s two receptors, M1 and M2 [272,273,274]. M1, M2 agonism has wide and disparate effects in several body systems [275,276]. The half life of melatonin is several minutes, while ramelteon’s half life is ~1 h. Ramelteon has three to four times greater affinity for melatonin receptors M1 and M2 than does melatonin itself [271,272,273]. Indirect evidence links disruption of melatonergic signaling with EC [277].

A review of data collected prior to 2015 indicated that malignant cells frequently express melatonergic receptors across the common cancers [275]. Since then, data have continued to accrue showing the ubiquity of cancer cell expression of M1 and/or M2 receptors. Although most known for synthesis in the pineal gland, melatonin is also synthesized and secreted by cells in bone marrow, cerebellar Purkinje cells, eosinophils, gut, kidney, liver, mast cells, NK cells, retina photoreceptors, T lymphocytes, uterus, and elsewhere [274]. Astrocyte melatonin synthesis is continuous and non-diurnal. Light exposure inhibits pineal melatonin secretion, and dark stimulates production.

The two most compelling rationales for adding ramelteon to anyone diagnosed with EC are 1) the data of Grin et al., who showed that average plasma melatonin level was 6.1 pg/mL in those with EC and 33.2 pg/mL in a risk matched control group without cancer [277], and 2) the data of Osanai et al. showing that ramelteon inhibited growth and invasiveness of estrogen receptor-positive EC cells [278].

In several different experimental animal models of EC, melatonin inhibited tumor proliferation and metastasis [279,280,281,282,283]. An interesting study by Kanishi et al. showed that in vitro inhibition of EC growth was restricted to EC cells that were positive for the 17-beta estradiol receptor [282]. In vitro melatonin decreased EC cell 17-beta estradiol receptor expression and 17-beta estradiol induced epithelial-to-mesenchymal transition [281,283]. These findings could reflect the activity of the IL-6 feedback cycle depicted in Figure 3 and discussed in Section 2.2.3. on celecoxib and IL-6.

## 3. Non-Pharmacological Interventions

Several non-pharmacological interventions, while not proven to benefit in EC5, have good epidemiological indications that they may reduce risk of EC recurrence [284].

### 3.1. Muscle Mass

Exercises designed to increase and maintain muscle mass may help. Those with an EC diagnosis who lose muscle mass have an average survival somewhat shorter than those who maintain or increase their muscle mass [285].

### 3.2. Diet

Maintain a plant based, predominantly vegetarian, low-fat diet. This need not be strict, however [286,287,288].

### 3.3. Meticulous Dental Hygiene

Poor dental hygiene results in low grade chronic upregulation of systemic cytokines that have potential to further cancer growth generally and EC growth specifically [289,290,291].

### 3.4. Sleep Hygiene

Maintain absolute dark conditions at night. Assure sound 9–11 h sleep. Avoid caffeine after noon if there is any evidence of sleep problems. Maintain a vigorous exercise program most days of the week. If medical help for sleep becomes necessary, use mirtazapine [292,293,294].

#### EC, NLR, Tadalafil, and TICO

The elevation of the ratio of neutrophils to lymphocytes (NLR) in peripheral blood is an underappreciated sign and concomitant of active cancer [295,296,297,298], seen also specifically in EC [299,300,301,302,303,304,305,306,307]. TICO is an abbreviation of a four repurposed drug regimen designed to bring an elevated NLR down to a more favorable level [297]. TICO uses tadalafil, a drug to lower pulmonary hypertension; an acne treatment, isotretinoin; colchicine, used to prevent gout; and omega-3 fatty acids (fish oil) [297].

As EC progressively invades the myometrium, the NLR increases, regardless of EC subtype or malignancy grade [301,308]. Other studies showed a similar increase in NLR as myometrial invasion increased, but also found higher NLR in higher grade EC [307]. In EC, both serum IL-6 [140,207] and EC cell COX-2 expression [141,185] also increase progressively as the myometrium is progressively invaded by EC.

In EC cases with elevated NLR, the total leukocyte count tends to be within the normal range [306]. Particularly instructive, the progressive NLR increases paralleled CA-125 increases in endometrial pathology, from

- endometrial hyperplasia without atypia, NLR 1.5 and CA125 5.0 U/mL,
- to endometrial hyperplasia with atypia, NLR 2.4 and CA125 12.0 U/mL,
- to EC with                                                  NLR 3.7 and CA125 54 U/mL [308].

The conclusion of this NLR data in EC is that the NLR should be followed in people trying EC5. If measures to identify the origin of a NLR > 3 do not give a remediable origin, elements of TICO can be added.

## 4. Discussion and Caveats

As in the aphorisms of the Preface to this paper, when embarking on any medical intervention, one faces some risk of net harm, however small. The difficult task of physician and patient to evaluate the balance and decide how to proceed becomes particularly difficult when considering the repurposed regimens of which I write, including the EC5 here. The database on which EC5 is based was orthodox, well established science and physiology-based principles. But only a formal trial can confirm the value of the EC5 regimen.

The primary caveat concerning the EC5 regimen is the common phenomenon of drugs showing strong preclinical cancer inhibition failing to do so in their clinical trials.

Cancer treatments generally tend to be more successful when the total body’s tumor mass is smallest. This is a generality, not a rule. This generality holds for repurposed augmentation regimens like EC5.

As in the message of the Preface to this paper, efforts to avoid any risk of harm can be harmful themselves.

A common problem when using celecoxib is underdosing. Pain can be treated with under 400 mg/day but, on theoretical grounds, cancer treatment should start at 400 mg twice daily [128].

Some elements marshalled to support the use of the EC5 drugs in EC were only based on a few studies. Ideally, we should make medical treatment plans based on data that have been replicated many times. We do not have this for most elements regarding the rationale for EC5.

On the other hand, “we go to war with the army we have, not the army we would like to have” (D. Rumsfeld, USA Secretary of Defense, 2001–2006).

Much of the work on alendronate inhibition of cancer growth was published one or two decades ago. Why is alendronate not commonly used in cancer treatment beyond its bone protection role? The reviewed data here and in Table 3 would seem to warrant further clinical study, particularly in combination with simvastatin.

None of the clinical studies amplifying gamma–delta T cell numbers or function have yielded positive results. Why they have not is unknown.

It is crucial to have 24/7 immediate phone access to the prescribing physician. If this condition cannot be met, any unproven repurposed regimen should be avoided.

## 5. Conclusions

The EC5 regimen has a sound rationale for adjunctive use in treating EC but proof of efficacy or safety is lacking. Until we have such proof of safety and efficacy, the decision to use part or all EC5 rests with the individual doctor and the patient’s own assessment of the benefit/risk ratio.

## Figures and Tables

**Table 1:** List of EC5 drugs with their general medical use and proposed use in EC, and their direct and indirect effects. The listed general uses and use in EC are representative, not comprehensive. Hh, Hedgehog signaling; MMP, matrix metalloproteinase; M1, M2, melatonin receptors. The listed “use in EC” is representative, not comprehensive. For all the EC5 drugs, the core rationale rests on the empirical demonstration of growth inhibition in the preclinical study.

Drug	General Use	Use in EC
alendronate	osteoporosis	empirical
celecoxib	analgesia	inhibit CA-IX,-XII, COX-2, IL-6
itraconazole	fungal infection	inhibit Hh, Wnt, P-gp,
ramelteon	sleep aid	M1, M2 agonism
simvastatin	hypercholesterolemia	augment alendronate

**Table 2:** EC5 drugs, target doses, and common side effects. Most people starting these medicines will experience none or only minimal and well-tolerated side effects. Serious adverse events are rare but possible and not listed here. Notable among those rare serious adverse events are osteonecrosis with alendronate, rhabdomyolysis with simvastatin, and fulminant hepatitis with itraconazole.

Drug	Target Dose	Common Side Effects
alendronate *	30 mg × 1/week	upper GI irritation, osteonecrosis rare but serious
celecoxib	400 mg bid	none are common
itraconazole	200 mg bid	minor LFT elevation, high potential for drug-drug interaction
ramelteon	8–16 mg × 1 hs	dysgeusia, day fatigue
simvastatin	40 mg × 1	HA, minor GI disturbance

* Alendronate must be taken 1 h before the first food or drink of the day and people must remain seated upright for 1 h after ingestion. HA, headache; LFT, hepatic transaminases. Many potential side effects of these drugs are not listed here.

**Table 3:** Some representative references of evidence that alendronate can inhibit cancer growth across a wide variety of cancer types.

AML	[41]
breast	[42,43]
chondrosarcoma	[44]
colon	[45]
endometrial	[32]
fibrosarcoma	[46]
gastric	[47]
giant cell tumors	[48]
glioblastoma	[22]
hepatocellular	[49]
melanoma	[40]
myeloma	[50]
osteosarcoma	[51]
ovarian	[32]
pancreatic	[42]
prostate	[42]

## Data Availability

The published paper contains all relevant data.
